# Research progress on the diagnostic value of fecal calprotectin in colorectal tumors

**DOI:** 10.3389/fmed.2025.1626197

**Published:** 2025-07-09

**Authors:** Wei Jing, Jiangshan Sun, Qingzheng Wu, Xiuli Zhang

**Affiliations:** ^1^Microbiota Division, Department of Gastroenterology and Hepatology, The First Medical Center of Chinese People’s Liberation Army (PLA) General Hospital, Beijing, China; ^2^School of Medicine, Nankai University, Tianjin, China; ^3^Department of Endocrinology, The First Medical Center of Chinese People’s Liberation Army (PLA) General Hospital, Beijing, China

**Keywords:** fecal calprotectin (FC), colorectal tumors, inflammation, diagnostic, non-invasive biomarkers

## Abstract

Fecal Calprotectin (FC), a calcium-binding protein secreted by neutrophils and macrophages and belonging to the S100 protein family, has gained increasing utilization in recent years for the diagnosis and monitoring of intestinal diseases due to its high expression and stability in inflammatory responses. In the field of colorectal tumors, the diagnostic value of FC has gradually emerged. Within colorectal tumors, colorectal cancer (CRC) is an area of significant research focus. Studies have demonstrated significantly elevated FC levels in patients with CRC, a phenomenon potentially linked to chronic inflammation and immune cell infiltration within the tumor microenvironment. FC exhibits notable advantages in colorectal tumor diagnosis, characterized by high sensitivity and moderate specificity. Emerging research has revealed correlations between FC levels and colorectal tumors staging as well as left-sided versus right-sided tumor localization, with elevated FC levels associated with malignant transformation, local inflammation, and advanced tumor stages (T3 and T4). The diagnostic performance of fecal calprotectin (FC) as a non-invasive marker for colorectal tumors has not yet been established. However, due to its association with inflammation, FC holds promise for playing a more significant role in the screening, staging, and localization of colorectal tumors.

## 1 Introduction

Colorectal cancer (CRC) is currently the third most common malignant tumor and the second leading cause of cancer-related deaths (1). By 2035, the global annual incidence of CRC is projected to rise to 2.5 million (2). Most CRC cases follow the carcinogenesis sequence of “adenoma → low-grade intraepithelial neoplasia → high-grade intraepithelial neoplasia → carcinoma” (3), a process that is relatively slow and uniform, providing a critical window for prevention, early diagnosis, and treatment. The overall survival rate for advanced CRC is low, with a 5-year survival rate of only 50% (4).

The prognosis of CRC largely depends on early diagnosis (5). Although colonoscopy is the primary method for diagnosing and detecting CRC or early-stage lesions, its specialized nature, invasiveness, and non-negligible risk of complications (6) make it unsuitable for large-scale population screening. Therefore, there is a need to identify non-invasive biomarkers suitable for CRC screening (7).

Currently, the “two-step” strategy—initial fecal immunochemical testing (FIT) followed by secondary colonoscopy for positive cases—remains the most widely adopted CRC screening approach globally. However, to date, no marker with 100% sensitivity and specificity for CRC has been identified. Fecal calprotectin (FC), a calcium-containing antimicrobial protein complex released from neutrophils during acute and chronic intestinal inflammation, is currently a widely used biomarker for diagnosing and monitoring inflammatory bowel disease (IBD) (8). But does FC have diagnostic value for intestinal tumors? Existing research on this topic is insufficient, and its diagnostic role in CRC remains inconclusive. This article reviews the research progress on the diagnostic value of FC in colorectal tumors.

## 2 Fecal calprotectin—an inflammatory non-invasive biomarker

Fecal calprotectin (FC) is a calcium-containing antimicrobial protein complex that accounts for 60% of cytoplasmic proteins in neutrophils. It is released during acute and chronic inflammation of the gastrointestinal wall and serves as a sensitive indicator of colonic inflammation, primarily used for the clinical assessment of IBD (9). However, FC’s role is not limited to a single disease; elevated FC levels occur in various gastrointestinal disorders, including colitis and malignancies (10). FC constitutes approximately 60% of the soluble proteins in human neutrophil cytoplasm and is also present in monocytes, macrophages, and ileal tissue eosinophils (11). In response to inflammatory stimuli, it is excreted into the intestinal lumen, resulting in fecal concentrations six times higher than in plasma and other bodily fluids (12).

FC belongs to the S-100 protein family and consists of three polypeptide chains, primarily found in the cytoplasm of neutrophils and the membranes of monocytes (9, 13, 14). The S-100 protein family comprises over 20 known calcium-binding proteins with tissue-specific expression patterns. S100A8, S100A9, and S100A12 are specifically linked to innate immune function through their expression in phagocytes (15). FC is released upon neutrophil death or injury (16) and is believed to modulate components of the inflammatory process (9, 17). The S100A8/S100A9 complex regulates intracellular pathways in innate immune cells, coordinates inflammatory responses, facilitates leukocyte aggregation, and promotes the transport of arachidonic acid to inflammatory sites (18). Beyond its role in acute inflammation, FC also controls cell proliferation, differentiation, and apoptosis. Recent studies suggest that FC drives inflammation beyond mucosal surfaces. For example, S100A8/A9 regulates the tumor microenvironment in various cancers, activating triggers for tumorigenesis (19), and S100A9-deficient mice are protected from intestinal tumorigenesis and inflammation (20). FC’s involvement in inflammation and tumors endows it with unique biological characteristics, potentially making it valuable for diagnosing colorectal tumors.

There is no universally defined normal value for FC. The earliest detection method was the enzyme-linked immunosorbent assay (ELISA) developed by Roseth et al. Later, enzyme immunoassay (EIA) methods emerged, with sensitivities five times higher than ELISA (units: μg/g). Most researchers define the normal FC value as 50 μg/g (21), while others consider it to be 200 μg/g (10). Organizations such as the British Society of Gastroenterology (22) and the European Crohn’s and Colitis Organisation (23) have not clearly defined a “normal” FC value, recommending instead that trends in individual patient FC levels be used as guidance. Therefore, further large-scale clinical trials are needed to establish the normal range for FC.

FC is currently a widely used biomarker for diagnosing and monitoring IBD (8). In adults, an FC level >50 μg/g has a sensitivity of 93% and specificity of 94% for distinguishing irritable bowel syndrome (IBS) from IBD (24). International guidelines recommend FC testing for IBD patients to assess disease activity and guide treatment (25). Although FC is easy to use and widely adopted, it is not specific to IBD. Other gastrointestinal conditions, such as nonsteroidal anti-inflammatory drug (NSAID) use and malignancies, can also elevate FC levels (26–28). Based on the above biological characteristics, FC may demonstrate certain value in early screening and prognostic evaluation of colorectal tumors by reflecting inflammatory status.

## 3 Colorectal tumors—inflammation

CRC can be classified as sporadic or hereditary (29). It is caused by somatic mutations that accumulate over a lifetime, with many environmental factors contributing to these mutations. Mutations in the adenomatous polyposis coli (APC) gene are considered the critical first step in the formation of benign adenomas. Healthy intestinal epithelial cells can develop into adenomas within 5 to 10 years, which may then transform into malignant cells (30). In addition to genetic factors, certain lifestyle choices (e.g., sedentary behavior, smoking, alcohol consumption, obesity, excessive red meat intake) and lower socioeconomic status are favorable factors for CRC development, while exercise, high-fiber diets, dairy products, and fish are protective measures (31).

The pathogenesis of CRC is divided into three types. The first is chromosomal instability (CIN), characterized by structural chromosomal abnormalities, aneuploidy, deletions, and rearrangements (32). Carcinogenesis via the CIN pathway involves chromosomal aberrations and mutations in genes such as APC and TP53 (32, 33). The second is microsatellite instability (MSI), which is associated with defects in the DNA mismatch repair (MMR) system, including inactivation of MLH1, MLH3, MSH2, MSH3, MSH6, or PMS2 genes (34). The third classification is the CpG island methylator phenotype (CIMP). DNA methyltransferases initiate methylation, particularly in tumors, thereby suppressing tumor suppressor genes and promoting CRC development (35, 36).

Intestinal inflammation can drive carcinogenesis, and the presence of an inflammatory microenvironment is a key hallmark of cancer (37). At the intersection of the two pathways (endogenous and exogenous) through which inflammation promotes cancer formation, key coordinating factors include transcription factors and major pro-inflammatory cytokines. Inflammation is a critical component of tumors and may represent the seventh hallmark of cancer (38). Factors mediating inflammation-driven tumor progression include transcription factors, cytokines, chemokines, and infiltrating leukocytes. Inflammation downregulates MMR proteins through various mechanisms, destabilizing the cancer cell genome and leading to the accumulation of genetic alterations, thereby contributing to MSI. In a study by Park et al. (39), it was noted that in the TNM staging of CRC, higher T stages were associated with lower immune scores and higher systemic inflammatory responses, indirectly indicating that inflammation is a key component of the tumor microenvironment, promoting cancer formation and progression.

## 4 Fecal calprotectin—colorectal tumors

### 4.1 Diagnostic efficacy of fecal calprotectin in colorectal tumors

Since Roseth et al. (40) first published research on FC and CRC in the early 1990s, the potential relationship between FC and CRC has garnered attention. In recent years, FC has shown potential value as a non-invasive biomarker for diagnosing colorectal tumors, including CRC and precancerous lesions (41). In colorectal tumor tissues, FC expression may be associated with tumor-associated macrophages (TAMs) and the tumor microenvironment (42).

Some researchers worldwide hold a positive view of FC’s diagnostic value for colorectal tumors. FC is released during inflammatory responses by neutrophils, macrophages, monocytes, and epithelial cells. Increased neutrophil shedding into the intestinal lumen makes FC more sensitive than fecal occult blood for identifying CRC patients (43). CRC patients exhibit higher FC levels than healthy individuals and patients with other types of tumors, making FC a sensitive marker for CRC (44). In a 2022 meta-analysis by Atefeh et al. (45), the diagnostic accuracy of FC for CRC was evaluated. Among 23 studies using ELISA to detect FC, the pooled sensitivity was 85%. This study also evaluated FC’s diagnostic accuracy for advanced adenomas for the first time, concluding that FC is a qualified biomarker for CRC detection, with covariates such as age, gender, measurement methods, and tumor location having no significant impact on its performance. In the 2016 study by Turvill et al. (46) involving 654 symptomatic patients suspected of having CRC, results showed that at a cutoff of 50 μg/g, FC had a negative predictive value (NPV) of 98.6% for CRC. When including polyps larger than 10 mm, the NPV was 97.2%, suggesting that FC could serve as a qualified CRC screening tool. In the 2022 retrospective study by Nathalie Blad et al. (47) involving 124 CRC patients, 98 (79%) had FC levels ≥50 μg/g, indicating high sensitivity for CRC diagnosis. In the 2019 study by Verma et al. (48) involving 1,919 patients, FC performed similarly to or better than FIT in a “low-risk” cohort of individuals under 50 years old, contrasting with previous studies that generally found FIT to be more sensitive than FC for CRC diagnosis (49, 50). In the 2022 study by Fiona et al. (51) involving 352 FIT-positive patients, at a cutoff of 50 μg/g, FC demonstrated a sensitivity of 92.8% and specificity of 41.7% for CRC diagnosis. Sensitivity increased progressively from non-advanced to malignant tumors, with the highest sensitivity observed for malignancies.

As FC’s diagnostic efficacy for colorectal tumors becomes increasingly evident, domestic researchers have also conducted studies. In a 2021 prospective study in China (52) involving 181 patients undergoing FC testing, 49 (27.07%) were diagnosed with CRC. Results showed that CRC patients had significantly higher FC levels than non-CRC patients, and FC levels increased with the severity of colorectal lesions. The sensitivity and NPV of FC combined with fecal occult blood testing for CRC diagnosis were significantly higher than those of FC or fecal occult blood testing alone.

However, while some studies hold a favorable view of FC for diagnosing colorectal tumors, others have refuted its diagnostic performance for these lesions. Ye et al. (53) published a meta-analysis on the diagnostic accuracy of FC for screening CRC, reporting that FC is not recommended for CRC detection. The 2020 study by Alexandre et al. (54) found that the colorectal mucus layer (CM) is the primary reservoir for biomarkers released by the colorectal mucosa. Comparing the diagnostic efficacy of nine CM proteins, FC showed a sensitivity of 57.5% and specificity of 80%, leading the authors to conclude that FC is not a reliable diagnostic marker. Evidence for FC’s diagnostic rate in colorectal tumors is less robust than for FIT, with significant heterogeneity and variability in sensitivity across studies. Some authors suggest that FC may be a more sensitive but less specific biomarker for CRC diagnosis compared to FIT (10). Ross et al. (10) pointed out that the sensitivity and specificity of FC for diagnosing CRC exhibit an inverse relationship. This low specificity hampers its utility as a practical screening tool for CRC diagnosis. However, its use in combination with other biomarkers holds potential as an adjunct for CRC screening (55). When FC is combined with FIT, existing research yields inconsistent results. While some studies suggest that the combination does not improve diagnostic accuracy compared to FIT alone (49, 56, 57), others report enhanced performance (49, 58). In the 2023 study by Gonzalo et al. (59) involving 571 patients, 118 (20.7%) were diagnosed with significant pathology, including 30 CRC cases. The NPVs of FIT, FC, and their combination for diagnosing significant pathology were 88.4, 87.6, and 90.8%, respectively, suggesting that combining FC with FIT may improve specificity. In a 2020 prospective study by Lué et al. (50), 404 patients were enrolled, among whom 87 had clinically significant pathology (including 16 CRC cases and 39 advanced adenomas). The NPVs for FC and FOBT were 90.1 and 86.9%, respectively. The combination of FC and FOBT yielded an NPV of 94.1%, with a sensitivity of 88.5% and specificity of 50.3%. These results demonstrate that the combination of FOBT and FC provides better diagnostic accuracy than either test used alone. In the 2017 study by Widlak et al. (56), which included 430 patients, the results showed similar NPVs (99%) for detecting colorectal cancer using FIT alone or the combination of both markers (FIT and FC). The sensitivity and specificity were 84 and 93%, respectively. Collectively, these studies suggest that FC alone may not be recommended for diagnosing colorectal tumors. However, when combined with other potential biomarkers (such as FOBT or FIT), it may improve diagnostic performance for colorectal tumors and could serve as a future adjunctive screening tool.

Based on the synthesis of the above studies, although FC demonstrates high sensitivity and NPV in colorectal tumor screening, particularly for exclusionary diagnosis, its specificity is compromised by factors such as IBD. Combining FC with other screening methods (e.g., FIT and colonoscopy) may improve diagnostic specificity. Additionally, as colorectal tumor risk assessment models evolve, FC could become part of such tools, offering more non-invasive screening options for symptomatic and screening populations.

### 4.2 Correlation between fecal calprotectin levels and colorectal tumors staging

After CRC formation, the local inflammatory microenvironment can promote the accumulation of mutations and epigenetic changes (37). Therefore, monitoring inflammatory responses may help track tumor progression, though insufficient evidence exists to establish a clear correlation between FC and CRC tumor size or staging. A 2016 Chinese case–control study (60) enrolled 246 CRC patients, 246 non-cancerous polyp patients, and 246 normal controls, analyzing FC levels across Dukes stages. The results demonstrated significantly elevated FC levels in both the non-cancerous polyp and CRC groups compared to controls. Specifically: Stage B showed markedly higher FC than Stage A; Stage C exceeded Stage A but remained lower than Stage B; Stage D was significantly elevated versus Stages A and C yet lower than Stage B. These findings indicate FC’s potential as a reference indicator for colorectal tumor staging. In a study by Raúl Y et al., FC was linked to CRC progression, potentially influencing tumor development rather than initiation (37). In the 2014 study by Lehmann et al. (61) involving 80 CRC patients (26 rectal, 29 left-sided, 23 right-sided, and 2 bilateral tumors), FC levels were significantly higher in T3 and T4 stages than in T1 and T2 stages, indicating that elevated FC is associated with malignant transformation, local inflammation (62), and advanced tumor stages (T3 and T4) (61).

### 4.3 Correlation between fecal calprotectin levels and left- vs. right-sided colorectal tumors

Although FC shows high sensitivity and NPV in CRC screening, few studies have examined its diagnostic efficacy for left- versus right-sided tumors. A 2016 Chinese case–control study (60) found that colon cancer patients (*n* = 109) had significantly higher FC levels than rectal cancer patients (*n* = 137) (205.36 μg/g vs. 126.84 μg/g), suggesting higher overall inflammatory burden in the colon, though left- and right-sided distinctions were not explicitly made. In the 2022 retrospective study by Nathalie Blad et al. (47), FC ≥ 50 μg/g was observed in 98 CRC patients (79%). The proportion of patients with FC ≥ 50 μg/g was significantly higher in those with right-sided CRC compared to left-sided CRC (92% vs. 74%, *p* = 0.027). In a binary logistic regression analysis, stage III/IV tumors (adjusted OR 3.47; 95% CI: 1.27–9.42) and right-sided tumor localization (adjusted OR 3.80; 95% CI: 1.01–14.3) were identified as independent risk factors significantly associated with FC ≥ 50 μg/g. These results suggest that FC levels are associated with colorectal tumor stage and the location of the tumor in the left or right colon, and that FC may potentially improve the detection rate for right-sided CRC.

Most studies have not clearly differentiated FC levels between left- and right-sided tumors, but right-sided colorectal mucosa may exhibit more pronounced inflammation, leading to higher FC levels. In a review by Baran et al. (63), differences between left- and right-sided CRC were explored. Right-sided CRC is characterized by high MSI and KRAS or BRAF mutations, whereas left-sided tumors exhibit greater chromosomal instability. Right-sided colorectal tumors may have more immunogenic features, theoretically leading to higher FC levels.

## 5 Conclusion

Colorectal tumors are a common malignant tumor in the digestive system, characterized by high incidence and mortality rates. However, its development process is relatively slow and unifocal, providing a crucial time window for early diagnosis and treatment. Colonoscopy with pathological examination remains the gold standard for colorectal tumors diagnosis, but its invasive nature reduces patient compliance. Consequently, non-invasive screening markers for colorectal tumors have become a major research focus in recent years.

Fecal calprotectin (FC) is a calcium-binding antimicrobial protein complex currently primarily used in clinical practice for the diagnosis and monitoring of inflammatory bowel disease (IBD). Intestinal inflammation can drive carcinogenesis and promote cancer formation and progression. Since FC reflects the state of intestinal inflammation, it may hold value in colorectal tumors screening. While some studies hold a favorable view of FC for diagnosing colorectal tumors, others have refuted its diagnostic performance. Therefore, the diagnostic efficacy of FC as a non-invasive biomarker for colorectal tumors remains inconclusive. Some research suggests that combining FC with markers like fecal occult blood testing (FOBT)/fecal immunochemical testing (FIT) may improve diagnostic accuracy.

Future large-scale clinical trials are needed to evaluate whether FC can serve as an adjunct to existing colorectal tumors screening methods and to assess the diagnostic performance of FC combined with these methods, including FIT/FOBT. Future research should also further validate the diagnostic efficacy of FC across different ethnic populations, clarify its relationship with colorectal tumors staging and localization, and explore its association with tumor molecular characteristics (such as KRAS mutations, BRAF mutations). Additionally, studies should investigate whether FC can rationalize the use of colonoscopy in asymptomatic screening populations.
